# Disruptive Communication as a Means to Engage Children in Solving Environmental Challenges: A Case Study on Plastic Pollution

**DOI:** 10.3389/fpsyg.2021.635448

**Published:** 2021-09-16

**Authors:** Erica Löfström, Isabel Richter, Ine H. Nesvold

**Affiliations:** ^1^Citizens, Environment and Safety (CES), Faculty of Social and Educational Sciences, Department of Psychology, Norwegian University of Science and Technology (NTNU), Trondheim, Norway; ^2^Faculty of Health, School of Psychology, University of Plymouth, Plymouth, United Kingdom

**Keywords:** children, environmental communication, disruptive communication, plastic pollution, co-creation activities, human and environment

## Abstract

Environmental degradation and how we care for our planet are some of the greatest challenges the world is up against at this moment. These challenges has received increased focus in both, research and the public sphere. So far, most of this attention revolved around adult’s attitudes and behavior. However, environmental engagement amongst the younger generation gains in popularity. Using plastic pollution as a case, this qualitative study aims to acquire insights into the mental models of children. We collected qualitative data during an innovative, structured workshop according to the “Nature In Your Face” (NIYF) framework. The approach challenges the assumption that the societal change can be achieved gradually, with non-invasive techniques. Instead, we explore the potential of disruption to push citizens out of their comfort zone, thereby making room for co-creation. The disruption was performed by confronting 36 fifth graders from a Norwegian primary school, with disturbing images of plastic contaminating their local shorelines. The data was obtained by using the workshop framework, combined with semi-structured group interviews. The interview data was analyzed by means of thematic analysis. We found that the disruptions triggered emotional reactions like anger and fear. With these emotions as a driving force, the first workshop step was introduced, the Framing of the problem. The next step, Twisting the problem, was reflected in the children developing their own, creative solutions and creatively engaged with them in groups. The last step, Using, was only touched upon in the workshop and is therefore beyond the scope of this paper. Our results indicate that there are three prominent themes reflecting how children discuss plastic pollution. The children talked about their (1) Emotions related to plastic pollution, (2) Attitudes related to plastic, and (3) Perceptions of plastic pollution. These themes were further subdivided into different types of emotions, characteristics of plastic as a material as well as perceptions on different locations of unnecessary plastic. Psychologically, the mechanisms underlying the identified themes were linked to eco-anxiety, denial, self-efficacy, and cognitive dissonance. We conclude that disruptive eco-visualization can create an emotional response amongst children, which can be transformed into co-creation of ideas.

## Introduction

We are currently finding ourselves in what the UN General Assembly calls the “Decade of action and delivery for sustainable development” (United Nations General Assembly, 2019). At the same time, we are experiencing a global inertia to act on environmental challenges (Hornsey and Fielding, 2020). Calls get louder for local initiatives on city and community level, which are recognized in their critical role in implementing and realizing the Sustainable Development Goals (United Nations General Assembly, 2019).

So far, countless strategies have been developed attempting to carefully and gradually nudge people toward a more sustainable way of life. Most of these strategies aim to raise awareness, strengthen environmental attitudes or increase motivation for sustainable behaviors (Feola, 2015). Examples for these strategies are prompts, social marketing, nudging, foot-in-the-door, door-in-the-face or reward and punishment (Cialdini, 2009; McKenzie-Mohr et al., 2011; Klöckner, 2015). Unfortunately, these strategies only lead to slow change and are prone to fail because of hardwired routines, salient social norms, situational factors or other “dragons” of inaction (Gifford, 2011).

With less than 10 years to meet greenhouse gas emission targets, predictions that more plastic than fish will be in the ocean by 2050 (Ellen MacArthur Foundation, 2016) and alarming rates of biodiversity loss (Mace et al., 2018), changes in our lifestyles are necessary immediately. We are currently facing a mismatch between the acknowledgment of environmental challenges and actual change processes to mitigate these challenges (Kollmuss and Agyeman, 2002; Fennis et al., 2011; Moser and Dilling, 2011; Sheeran and Webb, 2016). Immediate change can be elicited by disruptive forces which open windows of opportunity for change (Birkmann et al., 2010; Schäfer et al., 2012; Thomas et al., 2016; Verplanken and Roy, 2016). This disruptive force can manifest in many ways: natural disasters, dynamics in global leadership or diseases as we are currently experiencing with the COVID-19 pandemic (Ibn-Mohammed et al., 2020; Lambert et al., 2020; Shih, 2020). As a consequence people have to sharply adapt their way of life, and overcome habits, form new social norms and change behavior on a large scale. This change, however, is not necessarily in line with sustainable targets, often rather the opposite.

We believe that it is possible to artificially evoke disruption and thereby induce change processes. This is a novel approach and based on various strain of literature pointing toward the role of emotions as catalysts of action (Pooley and O’Connor, 2000; Salama and Aboukoura, 2018; Landmann and Rohmann, 2020) and the power of disruption as opportunity (or *window*) for change (Birkmann et al., 2010; Verplanken and Roy, 2016; Richter et al., 2021). The advantage of artificially induced disruption is, however, that we can channel the energy of change in a sustainable direction and thereby avoid the feeling of helplessness (Bamberg, 2002).

In this article we present an innovative approach, combining disruptive communication with creative engagement for sustainable change. We present qualitative data derived from a first pilot study trialing this approach. We then evaluate success and discuss potential modifications of the method before we conclude with future directions and implications.

### Power of Eco-Visualizations

In environmental communication, the use of visuals is one of the core recommendations (Nicholson-Cole, 2005; Sheppard, 2012; Moser, 2014; Corner et al., 2015; Klöckner, 2015) as it refers to the human preference of visual information processing. Visuals steer our attention, trigger strong emotions as compared to textual information (Gardner and Stern, 1996; E. A. Holmes and Mathews, 2010), are fast to process and cost-effective to implement (Pahl et al., 2016). Zlatev et al. (2010) even claim that visualizations provoke our motivation by activating relevant goals.

Applying these afore mentioned principles for environmental conservation, eco-visualizations are traditionally used to raise awareness or knowledge around environmental problems such as climate change (Löfström and Pettersen, 2011) or sustainable management of local environments (Richter et al., under review)^1^. In most cases, eco-visualizations make the invisible visible (Pahl et al., 2016); they show resource use in real time or showcase the amount of melting sea ice during the last century (Holmes, 2009; Sheppard et al., 2011).

Eco-visualizations can occur in many different forms. To make sure they are successfully transmitting their message, it is important to always consider four aspects namely the technology with which they are expressed, the physical context and location, the social context including social media and their potential to shape the future (through political discourse or public debate) (Löfström, 2008; Löfström and Svanæs, 2017). Previous examples for eco-visualizations are the power aware cord (Gustafsson and Gyllenswärd, 2005; Löfström, 2007), the mobile application Ducky (Löfström and Svanæs, 2017), the art project “7,000 oaks and counting” (Holmes, 2009) or the Pollution Pods (Sommer et al., 2019).

### Role of Emotions in Environmental Behavior

The gap between acknowledging environmental problems and doing something about them could potentially arise from a lack of emotional involvement according to Roeser et al. (2012). Emotions are the drivers for advocacy behavior that can result from facing climate change information (Nabi et al., 2018) and they account for a large part of variance explaining environmentally relevant behavior, together with cognitions (Pooley and O’Connor, 2000). The downside of emotions as behavioral drivers is that they are only temporarily and lose their power to change long-established behaviors when they ebb off (Schwartz and Loewenstein, 2017). Channeling emotions into solution development or collective engagement can perpetuate the dynamic (Landmann and Rohmann, 2020).

So far, research has not found consensus if positive or negative emotions are more effective in facilitating climate action. Negative emotions like fear and anger can work as catalysts for climate action as long as the emotions are neither too weak, not too overwhelming (O’Neill and Nicholson-Cole, 2009) and as long as the communication contains an element that guarantees a sense of self-efficacy (Tannenbaum et al., 2015; Nabi et al., 2019). However, Schoenmaker (2020) did not find evidence for fear appeals to be effective, with or without coping appraisals. Positive emotions such as hope can increase acceptance of threatening information, facilitate strategy development and mobilize resources for adaptation (Das and Fennis, 2008). Especially when the threat is overwhelming as it is for many environmental challenges, hope can act as a motivational force and should be instilled (Ojala, 2012). Positive emotions might, however obscure the need for change and prevent people from becoming active. Combining fear and hope strategically (Nabi and Myrick, 2019), for example throughout a workshop format, and thereby creating an emotional flow (Nabi, 2015; Nabi and Green, 2015), is a promising pathway.

### Nature in Your Face

The NIYF framework uses disruptive communication strategies as a means to evoke strong emotions which do not have to—but may well be—negative. These emotions are used as a catalyst for engagement and elicit self-efficacy via creative work on solutions as part of a three-step vision workshop structure; framing-twisting and using, which is led by trained researcher(s). Theoretically, the NIYF concept is built on social practice theory (Shove et al., 2012) which assumes that peoples’ behavioral practices are rooted in a complex interaction of physical structures, regulations, and attached meanings. Hence, NIYF does not aim to elicit change at a personal level, but to contribute to societal transformation Feola (2015). NIYF also builds on elements of social influence and group processes (Cialdini, 2003; Abrahamse and Steg, 2013). Furthermore, by introducing an initial disruption (eco-visualization) that is in line with what has been defined as an imposed transformation, we enable a group process that allows for a reconceptualization of some elements of the societal system (Folke et al., 2010; O’Brien, 2012) which contributes to active transformation. For a more detailed description of the theoretical framework please see the more conceptual paper by Löfström et al. (2020).

The rationale behind the framework is to use the emotional response during the vision workshop as an entrance point for co-creation (Dunne and Raby, 2013). Collaboratively working on solutions shall stimulate (long-term) engagement on the issue. The latter is not, however, explored in this study, but will be included in the further NIYF work. The collaborative focus on solutions during group work shall induce the feeling of hope and belonging which consequently elicits emotional flow from negative to positive emotions (Nabi and Green, 2015). This community spirit and flow can serve as a springboard for further studies and NIYF project activities, which will be part of the recently awarded funding from the Norwegian Research Council (NFR, project No 302111).

The different stages of the project have been defined to provide structure and standardization during this, still mainly exploratory, process. The first stage, *framing*, is meant to limit the scope and magnitude of the problem (in this case plastic pollution) and make it more manageable (Rosso, 2014). We did this by framing the problem geographically by giving the children a “room” in which they were able to brainstorm in small groups. For stage two, *twisting*, the groups were presented with a zero plastic challenge for their respective room. This represents creative stage of the methodology which shall elicit innovative, solution-oriented thinking (Gray et al., 2010). Twisting is introduced after framing the problem, hence after it has been made manageable, which enables the participants to comfortably explore the problem and take on the challenge of solving it. The last stage, *using*, is meant to take the solutions and ideas further, for example discuss them with local decision makers and eventually implement at least part of the ideas. However, in this study, this stage was only touched upon due to lack of resources and time. This will be explored in future studies and is the aspired outcome for the main NIYF project.

### Plastic Pollution as Focus Area

The historic area we find ourselves in at the moment is often referred to as the plastic age (Thompson et al., 2009). Despite increasing awareness of the negative environmental consequences, production and consumption of plastic are on the rise and with it, the amount of plastic ending up in the environment (Jambeck et al., 2015). Compared to other environmental problems such as climate change, plastic pollution is more visible (Anderson et al., 2016; Pahl et al., 2017). Despite the fact the plastic pollution in Norway is not as severe as it is in some other countries (Lebreton et al., 2017), it still is a challenge that needs addressing. We selected plastic pollution as a case for this study as it is a popular problem that is often discussed in the media (Henderson and Green, 2020), most people are concerned about it (Pahl et al., 2020) and it is relatively easy to create disruptive communication in form of visuals.

### Involvement of Children

Not much research has been done yet that involves children into the discourse around environmental problems. It is not only very likely that children are aware of environmental problems such as climate change and plastic pollution, they are also showing signs that they are confused by the magnitude of the threat, feel anxious or concerned (Fritze et al., 2008). Some studies have looked at how children handle information about climate change, and some studies looked at how children approach environmental problems and develop solutions (Desjardins and Wakkary, 2011; Banerjee and Horn, 2014; Hartley et al., 2015; Richter et al., under review, see footnote 1).

Banerjee and Horn (2014) show that children can learn and understand energy use through a game. Desjardins and Wakkary (2011) shows that children know a lot about sustainability even if they might lack the correct terminology, Hartley et al. (2015) and Richter et al. (under review, see footnote 1) show that children can develop creative solutions, and that this creative engagement itself might have significant effects on their motivation to act.

The relevance of involving children into the discourse about environmental challenges as well as into the development of solutions is crucial, not only because experiences like this potentially makes them environmentally conscious adults (Molinario et al., 2020) but also because we can learn a lot when we listen to the creative ideas provided by younger generations.

## Materials and Methods

### Sample

As the main interest of this project was to involve children into the discussion around plastic pollution, 36 fifth grade pupils from Kristiansund primary school were recruited via convenience sampling in January 2020. Kristiansund is a coastal community with a long and scattered shore line where plastic litter has started to become a visible problem. The children (15 boys, 21 girls) were between 9 and 10 years old and belonged to two different classes, together representing the full 5th grade of the school. For the workshop, eight groups of 4–5 children each were formed randomly.

### Study Design

The study design was guided by the NIYF framework consisting of an eco-visualization followed by the three phases framing, twisting and using as described before. With the help of their teachers, a 4 h workshop was conducted with the selected children. The split of work and break times was identical to the children’s normal school days in order to provide a familiar structure. The workshop was co-led by researchers (EL and IN) with the support of the ordinary school teachers.

Three simplified forms of eco-visualizations were presented to the children in form of photographs. The photographs represented three different cases of plastic pollution: two from the children’s local beaches polluted by litter (Edvardsen, 2018; Löfström and Klöckner, 2019), and one showing a turtle that is about to feed on a plastic bag (Mayne, 2020). Subsequently, the children were divided into eight groups. To save time, the group membership was randomly allocated by the teachers before the workshop.

The first step, framing, was realized by defining four different frames in which the children were supposed to work: their classroom, the grocery store, their bedroom and the school playground. Each group was assigned one frame, which results in two groups per frame.

The second step, twisting, was realized by presenting the children with a scenario in which all plastic production around the world would be stopped. Their task was to imagine how their frame would be affected and to come up with solutions under this vision. To facilitate creativity, every group received different colored paper, colored pens, glue, scissors, and magazines.

**FIGURE 1:**
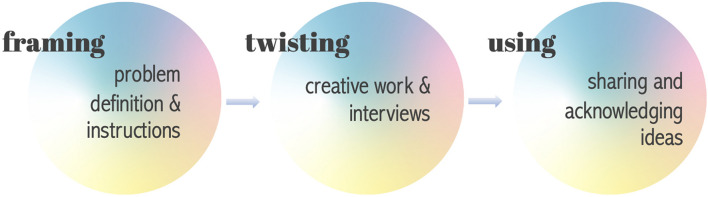
Illustration of the three interdependent steps of the NIYF methodology (by Löfström).

The third and last step, using, could only be partly implemented due to the lack of time and resources in this pilot study. Each group presented their results and a group discussion was held. All children received a diploma at the end of the workshop. The three interdependent steps are illustrated in Figure 1.

### Semi-Structured Interviews

With each of the eight groups, semi-structured interviews were conducted by the researchers (EL and IN) during the workshop. This method was supposed to help our participants to open up about their experiences and thoughts about plastic pollution (Mason, 2017; Howitt, 2019). The interviews also provided an opportunity to make sure the children engage with the theme or to answer open questions. The decision for a semi-structured interview was made to allow for flexibility and follow-up questions we did not anticipate beforehand. We interviewed the children within their groups to allow for interpersonal interaction and discussion as well as to give them a feeling of confidence which is particularly important in a children sample (Tjora, 2017).

The interview guide consisted of three parts which were (1) general knowledge about plastic pollution, (2) frame-specific questions about plastic pollution, and (3) thoughts on how the plastic problem could be solved. The group interviews were consensually recorded on an audio device to allow for content transcription later on.

### Data-Analysis

According to the guidelines for qualitative data analysis (Big Q) by Braun and Clarke (2006) the interviews were thematically clustered. This method of analysis allows for flexibility in data interpretation and combines deductive and inductive approaches. In the case of NIYF, the theoretical background is still in development and results in this study being partly exploratory and data driven and partly deduced from research produced by Desjardins and Wakkary (2011) and Banerjee and Horn (2014); as well as Löfström and Klöckner (2019).

The analysis process was started in February 2020 and followed the Braun and Clarke (2006) six steps for thematic data analysis. These six steps are (1) get to know the material, (2) generate codes, (3) find distinct themes, (4) evaluate themes, (5) decide for and define themes, and (6) write a report. Despite the guidelines sounding like a linear process, the practice is more circular during which the researcher moves back and forth between the steps.

To get to know the data, the audio recording was transcribed and the researchers familiarized themselves with the transcriptions by reading them through several times as well as taking notes. In step two, as many meaningful codes as possible were generated to ensure that no valuable parts of the data are omitted (Braun and Clarke, 2006). To generate the codes, we used the software nVivo 12. Step two resulted in identifying 60 distinct codes representing meaningful units. An example is illustrated in Table 1.

**TABLE 1:** Example for meaningful unites and their codes (translated from Norwegian by IR).

Meaningful unit transcription	Code
“For example am I scared when there is a lot of different weather, for example hale and snow, then I think of the climate and I am scared of the future and that the planet is destroyed. Then I think that this must have something to do with the plastic”	Climate change in relation to plastic litter
“We talk about discarding paper and plastic in the right bins and where they should not go… for example of plastic ends up in the paper bin, this is not right”	Correctly discard plastic waste

**FIGURE 2:**
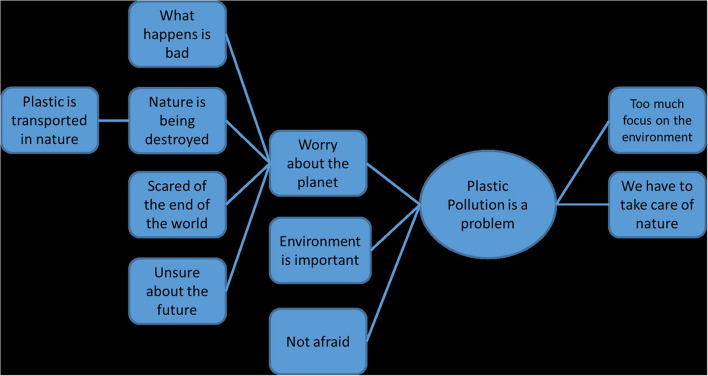
Example for a mind map created by nVivo 12 software (*translated from Norwegian by IR*).

In step three, the codes were synthesized into overarching themes with the help of nVivo 12 software which provided information of popular codes and codes that only appeared once and potentially could merged together. So were for example the codes “Unsure about the future,” “Scared of the end of the world” and “Nature is being destroyed” merged into the theme “Worry about the planet.” The codes with very low prevalence that could not be merged were taken out and saved into a separate folder. To visualize the relationships between codes, mind maps were created to connect the codes with each other and with the overarching theme. An example for a mind map can be found in Figure 2. Step three resulted in nine overarching themes, representing a varying number of subthemes and codes.

Step four consisted of thoroughly revisiting all themes and making sure the codes are internally and externally homogeneous. Furthermore, it was validated if the themes represent the dataset as a whole. The aim of this step is to conclude with a small number of themes that express the content of the dataset well. We concluded with three themes that represent the content of our dataset as well as the research question comprehensively. In step five each of the three themes have been defined and described in a separate document. Parts of step six, the wiring of a report, will be presented in the results section of this paper.

## Results

It is important to mention that the results presented here are subject to our interpretation, which was shaped by our theoretical framework, our research question and experience. The results section will be divided into two parts. First we will present the results retrieved via the thematic analysis of the semi-structured interviews. At this stage, we will provide some interview excerpts if relevant. All interview excerpts have been translated from Norwegian (IR). Subsequently, we will present the solutions the children developed during the disruptive communication workshop.

### Themes

The themes synthesized via the thematic analysis are presented in Table 2.

**TABLE 2:** Overview over themes synthesized from the semi-structured interviews.

Themes	Sub-categories
Emotions related to plastic pollution	• Anxiety • Frustration and helplessness
Attitudes related to plastic	• Negative characteristics of plastic as a material • Positive characteristics of plastic as a material
Perceptions of plastic pollution	• Unnecessary plastic • Plastic in grocery stores

#### Emotions Related to Plastic Pollution

The most apparent theme throughout the interviews was emotional reactions related to plastic pollution, mainly worry, anxiety, frustration and helplessness, hence, negative emotions. The theme was further subdivided into two categories which was worry and anxiety in relation to the future of the planet and frustration in relation to past generations, who caused the plastic pollution which the current generation has to deal with. In addition, many children emphasized as well how important it is to take care of nature and the environment.

The children explained that they are worried about the future of the planet if humanity continues the current course. Their worry relates to their own future on the one hand, but also to the future of the planet as a whole (Stine). Several statements evolved around the insecurity to predict future change due to plastic pollution, but also climate change and instability of the weather (Stine). Some informants even mentioned that they are afraid of the world being completely destroyed by humanity which would make it impossible to survive (Emilie). The children describe this worry like something that they are concerned about in everyday life and that causes stress (Emilie). Members of all six groups expressed clear feelings of worry and anxiety about the future, either during the interviews or during the creative engagement with the topic.

Stine: “This is destroying our future because we don’t know what will happen, what will happen in the future with all the plastic and the planet- I am actually a bit concerned about my future.” [int: You are concerned?] “yes because I am afraid that the world will die. No plants anymore and total chaos.”

Emilie: “Yes we need to prepare ourselves. And I am really stressed about everything…everything that might happen.”

Stine: “For example if there are many different kinds of weather, for example a little bit of snow and a little bit of hale, then I think about the climate and about that I am scared of the future and that the planet will be destroyed. And then I think that there is something about it, that the plastic has to be removed.”

The second subcategory evolves around the frustration toward past generations and the way they have been treating the environment. One of the informants’ mentions that what happens to the world is not his fault but the fault of past generations, but that his generation has to fix the problem (Stine). This can be interpreted as frustration about the current situation in which they have to take care of a problem they have not caused, something that is perceived as unfair (Anders). Some other informants explain that the attitude toward nature within past generations has been irresponsible, which they regard as not timely anymore (Helene). Despite the apparent frustration, the children still seem motivated to engage in solutions (Nora).

Stine: “It was our ancestors who have caused this, not us. But we need to clean it up when we are adults.”

Helene: “In the past it was like that that everything was fine and that it was not something stupid that they thought they can just throw their rubbish away and so on, but now this is not good anymore.”

Anders: “They just littered because they did not know what the plastic does so they just threw it everywhere. But now we know a lot…”

Nora: “Yes [motivated], we have been down near [location] and tidied up [litter]. The first time we drove to this place and then we came back after half a year and there was almost no rubbish anymore. But the first time we collected nine bags.”

#### Attitudes Related to Plastic

The second theme we identified was about the ways in which plastic was perceived, positive and negative. In psychology, these favorable or unfavorable evaluations of something or someone are described as attitudes (Fishbein and Ajzen, 1977). Throughout the process of analysis, we found that the children recognized positive and negative characteristics of plastic as material, which made it difficult for them to formulate a clear statement if plastic is ultimately good or bad.

**FIGURE 3:**
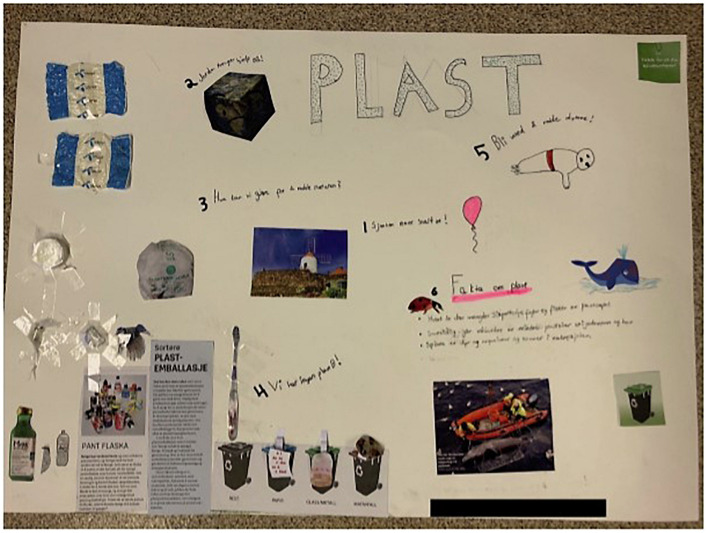
Example for outcome of creative engagement featuring negative consequences for animals.

The negative characteristics of plastic also included the consequences of its use and discard. One of the main statements the children made was therefore the danger discarded plastic poses for animals. Our participants seemed to be particularly concerned about animal welfare, which was reflected in the interviews (Anders, Helle) as well as in the creative engagement (Figure 3). The children describe the animals as innocent beings whilst humans are guilty of endangering them with their behavior (Tim).

Anders: “That we should not litter because then a deer could eat the plastic and then they can die.”

Helle: “That is really stupid because animals think it is food and then they eat it.”

Tim: “It is really bad that plastic harms them who live in the forest because they have been there first and then we come and destroy it.”

Other consequences of plastic pollution that were mentioned were water quality issues (Jenny), plastic in the ocean (Helle) as well as plastic particles in food (Tim).

Jenny: “And plastic ends up in the drinking water and then we drink the water and the food and drink is in danger.”

Helle: “Because if we litter the plastic goes down the hill on a rainy day and then the water brings it to the ocean.”

Tim: “Ehh we found out that we don’t know if there was plastic in the food we eat in some way.”

Characteristics of plastic that were mentioned was that it takes a long time to decompose which makes the consequences mentioned before even worse (Stine, Eline).

Stine: “Plastic takes… that it takes very long time to disappear in some way.”

Eline: “It takes a very long time to get rid of it.”

Despite the negative characteristic and consequences of plastic and plastic use dominated the discussions, some positive experiences with plastic have been mentioned, making plastic attractive as a material.

One characteristic that was mentioned as positive was how durable plastic is (Nora) and also how it helps to keep food edible for longer (Tim). Plastic also helped humanity to create new products for communication and travel (Tiril).

Nora: “Because it is a durable material and easy to use.”

Tim: “It makes it [food] keep for longer.”

Tiril: “Hmm it helped us to make a lot of things… like display protection of our mobile phones and things like that.”

Articulating positive and negative attitudes toward plastic at the same time turned out to be confusing for our participants. They seemed to struggle with the fact that plastic is difficult to avoid and that the positive characteristics might be used as an excuse for the negative ones.

#### Perceptions of Plastic Pollution

The last theme that emerged during the thematic analysis was perception of plastic consumption and pollution, especially in relation to the extent it is used for grocery packaging. Even the groups who did not work with grocery stores as their allocated frame did discuss food packaging and supermarkets. Perceptions are interpretation of sensory experiences which “enable organisms to organize and interpret the stimuli received into meaningful knowledge and to act in a coordinated manner” (APA Dictionary of Psychology, 2020).

The children perceive large parts of food packaging as unnecessary and hard to avoid (Synne, Sophia). Some describe the attempt to cut down on plastic packaging as almost impossible (Marte, Tiril).

Synne: “Because there is… everything we buy is packed in plastic.”

Sophia: “Almost everything in the shop is wrapped in plastic.”

Marte: “Because there is plastic in almost everything, in some way there is plastic in everything.”

Tiril: “There is microplastic in everything.”

One child describes that it is hard for her to distinguish plastic from other materials sometimes (Stine).

Stine: “Ehhh Barbie dolls for example! Their hair looks like real hair but it is plastic.”

The children also mention that they think that plastic consumption became a regular component of everyday life (Karoline). Ever since plastic was invented, the production and consumption escalated quickly and humanity became dependent on the material (Emilie). The children point out that changing habits connected to plastic is one of the main challenges (Anders).

Karoline: “We don’t need everything but since plastic is a useful material we began to use it for everything and thereby it became part of our daily life.”

Emilie: “I think there was surely one person who started with for example making a plastic bag and then everyone thought: wow, this person made a plastic bag! And then everyone started to do the same and then they produced even more things and this went on and on all the time.”

Anders: “Because we depend on it. If we for example go to [supermarket name] and buy a lot of food we can… instead of a cotton bag… we can just take a plastic bag. And if we start doing this all the time we end up with lots of plastic bags.”

### Workshop Outcomes

The workshop consisted of three steps, the eco-visualization, the framing and the twisting. As this was a pilot study, the last step, using, was not fully conducted, due to time- and monetary limitations.

**FIGURE 4:**
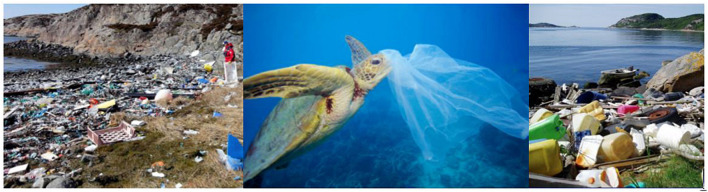
The eco-visualizations presented to all children at the beginning of the Nature in your Face Workshop [from left to right: Edvardsen (2018), Löfström and Klöckner (2019), Mayne (2020)].

The eco-visualization was composed of three pictures of plastic pollution (Figure 4). The children largely reacted with signs of shock and sadness. Especially the photograph of the turtle evoked strong emotional reactions. Similarly strong were the reactions, however, as the participants were informed that the two other pictures were taken in their local area, something they did not expect. When the pictures were shown, the children started having vivid conversations between each other, started asking questions and made comments.

Every group was allocated a certain topic to focus on (framing) and afterward received an identical twisting instruction and similar materials for the creative engagement. All the groups correctly adhered to their frame [classroom, grocery store (Figure 3), bedroom, and school playground] and most children understood the instructions for the twisting (“Imagine a world in which all plastic production and consumption is stopped, how would that look like in your frame?”) well.

Each group developed its own creative scenario, featuring distinct solutions and ideas. These between-group-differences demonstrate that the NIYF method evokes creative processes, even under identical frames. As an example, the groups who both worked with school playgrounds as their frames, focused on two different issues: One group presented solutions for cutting out plastic in various sportive activities like soccer and fetch; the other group elaborated on an efficient recycling infrastructure to avoid plastic litter (Figures 6, 7).

Some of the groups did collaborate better than others, coming up with more elaborate solutions. This points toward to importance of functioning social interactions for creative engagement and mutual inspiration in which one idea leads to another (Helene, Pia).

Helene: “In the grocery store the tomatoes could just be in a big basket and then you can bring your own basket and take them and place them in your own basket.”

Pia: “Yes! And what you can also do is that when you are in the supermarket usually when you are done you get a bag, but instead I think you can get your own personal shopping cart that you bring every time when you go to the shop.”

The most popular topics that were discussed across groups were exchanging plastic with alternative materials like paper, cardboard, glass, or wood (Stine, Eline) as well as raising awareness for more sustainable behaviors like reducing plastic consumption, recycling or clean-ups (Tilda, Tiril).

Stine: “We can for example instead of a plastic bag for example take all the oranges and fruit and use a paper bag.”

Eline: “Yes, just replace the plastic bag with a paper bag or cotton bag.”

Tilda: “That you can try to not shop more than you need. For example if you already have it from last time you don’t need to buy it again.”

Tiril: “It [clean-ups] took place a couple of times and sometimes with the clean ups you get a few hundred krona per bag.”

(see Figure 5).

**FIGURE 5:**
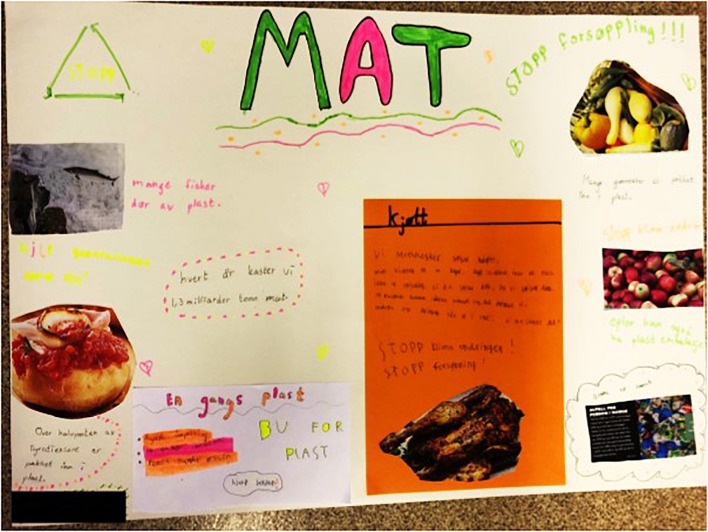
Example of creative engagement with the frame “grocery store” (*mat* means food in Norwegian).

**FIGURE 6:**
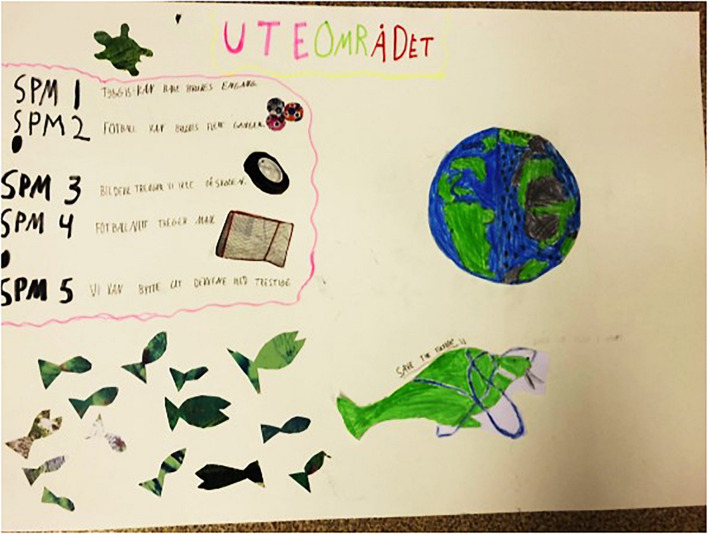
Creative engagement within the frame “school playground” illustrating different means of cutting out plastic in sportive activities (top left).

**FIGURE 7:**
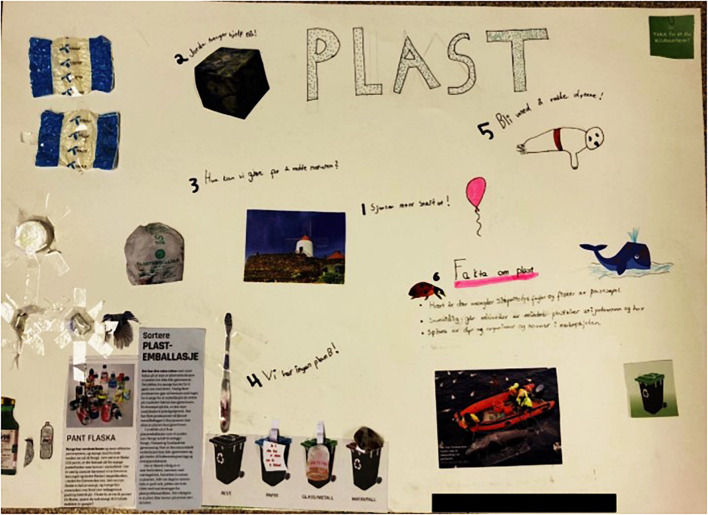
Creative engagement within the frame “school playground” illustrating the importance of recycling and different types of bins (bottom half).

Some children seemed to have difficulties with the twisting instruction and needed concrete explanations and support. This seemed to impede their creativity as they stayed very close to the given support and consequently did not think out of the box as much. In hindsight, this issue could have been avoided by conducting the interviews after the workshop.

All groups presented their posters in plenum explaining the solutions they came up with and discussing it with their peers in regards to feasibility and attractiveness. This can be understood as a pre-stage to the third step, using, that belongs to the NIYF approach. Some groups were more comfortable with speaking in front of their classmates than others, which had an influence on the quality of the subsequent discussion. Also during this stage, questions posed by the researchers seemed to put a damper on the children’s creativity and made them more focused on answering the questions correctly than on sharing their thoughts. In the NIYF rationale, using represents the step in which policy makers or community leaders take up the suggestions related by the workshop and bring it into action. This last step will be conducted in future NIYF workshops implementing the lessons learned from this pilot study.

## Discussion

### Underlying Psychological Mechanisms

The results of this study point toward three overarching concepts being at the core of how children experience plastic pollution namely (1) Emotions as a reaction to plastic pollution and evoked by the eco-visualization, (2) Attitudes toward plastic as positive and negative material, and (3) Perceptions of the extent of plastic pollution. We would like to discuss the underlying psychological mechanisms these emotions, attitudes and perceptions are pointing at and shed more light on how children experience plastic pollution.

**FIGURE 8:**
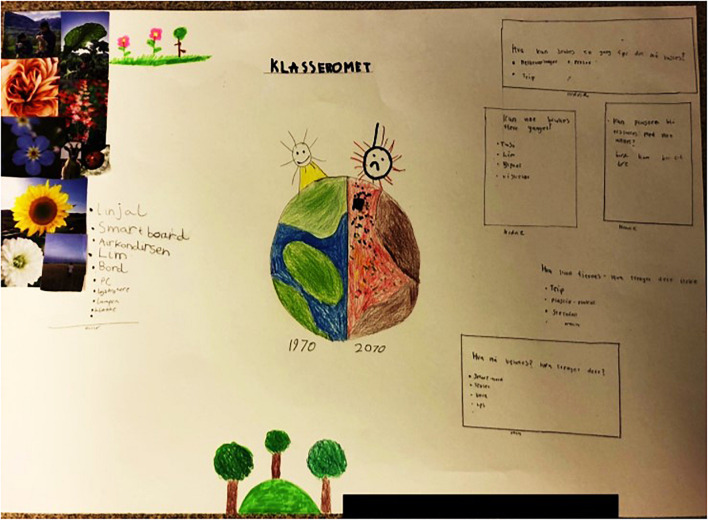
Creative engagement within the frame “classroom” illustrating, amongst other things, a drawing of a destroyed future planet.

Emotions of worry and frustration have been standing out during the data analysis. These emotions have not only been mentioned in connection to plastic pollution but also related to general environmental problems such as climate change. This type of negative emotions are part of a phenomenon called eco-anxiety (Carville, 2015; Clayton, 2020) which has been on the rise during the last century (Taylor and Murray, 2020). Lifton (2017) argues that high levels of eco-anxiety makes deterioration and destruction salient and thereby reminds people of their own death (Panu, 2020). Eco-anxiety amongst children has been noticed ever since environmental problems became part of the public discourse (Hutchinson, 1997; Hicks and Holden, 2007). Amongst our participants, many mentioned thoughts about the world coming to end and not being a safe living space anymore. Especially people who experienced natural disasters themselves often have high levels of eco-anxiety (Bourque and Cunsolo Willox, 2014; Clayton et al., 2017). Our participants have not experienced natural disasters but they have most likely been exposed to sensationalist media reporting on catastrophic events. Media reporting often makes use of sensationalist and shocking headlines as well as visual material that attracts attention. Children will potentially remember these headlines and images more clearly than written articles that might involve hopeful content as well (Nicholson-Cole, 2005; Holmes and Mathews, 2010; Weber, 2010). Children are not only more concerned (Burke et al., 2018), but also more affected by climate change as well as by future natural disasters (USGCRP, 2016). Many of our participating groups developed drawings showing how the world will look like in the future. Without exception, all these drawings have been gloomy (for example see Figure 8). This shows that even if children wish for their own future to be positive, they expect the world to change into a negative direction (Hicks and Holden, 2007). Negative emotions can function as catalyst for action if appropriate levels of self-efficacy are in place (Nicholson-Cole, 2005; Ojala, 2012). If self-efficacy is low, however, some people tend to use coping mechanisms to deal with overwhelming emotions such as distancing and denial (Tannenbaum et al., 2015). The concept of denial is considered an evolutionary defense mechanism employed from early childhood. Its goal is to aid adaptation by reducing anxiety and bolstering self-esteem (Cramer, 2006). Denial, or strategic downplaying of the seriousness of environmental problems has been observed in previous studies with children samples (Ojala, 2012). One of our participants described environmental problems as being exaggerated which he found frustrating, which could be interpreted as a form of denial. Stoknes (2015) argues that denial is the most popular strategy when people do not want to deal with environmental problems or need reasons not to adapt their own behavior. In children, denial has been found to be negatively associated with engagement and should be buffered with meaningful coping strategies (Ojala, 2012). We conclude that sufficient levels of self-efficacy are key for negative emotions to be translated into sustainable behavior change (Nabi et al., 2018).

Throughout the analysis, another dominant theme was the almost non-avoidable amount of plastic that is produced, consumed and discarded. Our participants often mentioned feelings of helplessness due to the sheer amount of plastic in the supermarkets, in their community and on the beaches. Plastic consumption, and thereby contributing to the problem, was described as hard to avoid which can, for some people, result in a low level of self-efficacy as well (Geiger et al., 2017). Low levels of self-efficacy related to the amount of plastic consumed can stem from people not having the knowledge and resources to reduce their plastic consumption. They experience that even if they try to cut down on packaging, plastic can be inside the product (Anderson et al., 2016) and often, they don’t know what to choose as an alternative. Our participants do not explicitly state that they lack the self-efficacy to reduce their plastic consumption. However, the combination of them discussing the large amount of plastic in everyday life, the difficulty to avid it and the negative emotions points toward low levels of self-efficacy when it comes to avoiding plastic.

Increasing self-efficacy can be achieved through various ways. Social modeling can be a way to increase levels of self-efficacy through observation of others who perform a certain behavior (Bandura, 1977; Bandura and Walters, 1977). Beach cleans, mostly performed in groups could be a way to get social modeling of relevant behaviors that help to cope with the plastic problem. However, social modeling could also have adverse effects in the context of plastic pollution as parents, peers or influencers might function as negative role models consuming large amounts of plastic (Ahuvia, 2015). Consciously choosing positive role models can be a way out of this dilemma. Beach cleans can also serve another function which is giving people a feeling of meaning, agency and social belonging (Wyles et al., 2017). We do not know yet, however, what returning to previously cleaned beaches and finding them littered again might do to the self-efficacy gained through the activity before. Workshop setups like NIYF can also be a way to increase self-efficacy. By collaborating with peers on finding solutions, workshop participants might feel like they are contributing to positive change (Bandura, 2002). At the same time, individual levels of self-efficacy can influence how participants engage in a workshop setup like NIYF. Low levels of self-efficacy might keep participants form active engagement which is down to their personal judgment of their abilities (Bandura et al., 1999).

The last theme we would like to discuss is the appreciation of plastic as not being exclusively negative. Despite the negative attributes of plastic being the dominant theme of the discussions, our participants also talked about positive aspects such as increased shelf life of products or innovations based on plastic (Zhang et al., 2014). This can potentially lead to a state called cognitive dissonance or, as Ojala (2007) calls it, attitudinal ambivalence. Cognitive dissonance is typically experienced as psychological stress because of mixed emotions because contradictory beliefs and actions are standing against each other (Festinger, 1962). Taking climate change as an example, so do knowledge and attitudes on sustainable lifestyles and mitigation often stand in contrast to people’s actual, unsustainable behavior (Stoknes, 2014, 2015). Striving for consistency, cognitive dissonance can be reduced by either changing relevant cognitions or relevant behavior. For the majority of people, adapting their cognitions is perceived as the easier option (Gifford, 2011; Taddicken and Wolff, 2020). For the young generation, cognitive dissonance has been found to be negatively associated with environmental behavior such as recycling (Ojala, 2007). However, as compared to negative attitudes, cognitive dissonance does not lead to ignorance but pushed people to action (as an example see Costarelli and Colloca, 2004), which, when channeled into the NIYF methodology can result in sustainable behavior change. As cognitive dissonance has not explicitly been measured in this study, we can only assume that the children experience it to some extent. Comments arguing that it is impossible to cut out plastic completely or that they recently participated in a beach clean and therefore did their part to help, point toward cognitive and behavioral adaptation strategies. As the NIYF framework is aimed at solution development and thereby building up agency and empowerment, we assume that cognitive dissonance might be reduced within our workshop participants.

### Success of the NIYF Workshop Structure

The results of the workshop indicate that the eco-visualization created active engagement and strong emotional reactions. This became clear during the group interviews, the children’s comments, social interactions and their facial expressions. The framing facilitated finding solutions within the children’s allocated context and all groups came up with different ideas, pointing toward high levels of creativity. The twisting turned out to work well for most groups but was perceived as difficult by some, indicating that the task descriptions could have been clearer or better formulated for the target group. The last step, using, has only been partly implemented due to limited time and resources and cannot be evaluated at this point.

Some groups showed highly creative solutions and strong engagement. These groups demonstrated that it is possible to use the NIYF framework to involve children in developing solutions for environmental problems. However, other groups had difficulties interacting with each other and therefore had a hard time with the task. This point toward to importance of functioning social interaction in order to be creative and develop feasible, innovative solutions. Trained facilitators to support constructive interactions, tailor-made instructions for the target group and pre-workshop teambuilding exercises to strengthen group coherence could help overcome this challenge.

In future workshops, the semi-structured interviews will be conducted after, not during the creative engagement. Although the interviews did indeed provide insight in the children’s thoughts and understanding of the plastic problem, but they also turned out to be a distraction and as a hindrance for creativity. However, the tight time-frame of this workshop may well have contributed to this problem, and not the questions *per se*. An alternative could be to have less questions during the engagement, and allocate more time for the tasks.

### Practical Implications

This study has shown how children can be actively involved in the discussion around plastic pollution and environmental conservation using eco-visualizations. In addition, we identified psychological mechanisms determining how children experience plastic pollutions.

For practitioners, this study provides implications regarding the workshop setup. A workshop like NIYF needs to be thoroughly planned and all communicative aspects need to be adapted to the specific target group. In our case, step two of the workshop, twisting, should have been introduced clearer to our participants to avoid confusion. Further, creativity should be least possible be interrupted by other tasks like interview questions. The quality of social interactions needs to be taken into account, especially when group work is part of the setup. As much as good teamwork can inspire creativity and boost productivity, malfunctioning groups can hinder these processes.

Regarding eco-visualizations, we can confirm that they work as tool to evoke emotions and disruption. It is paramount, however, to channel evoked emotions into a process of solution development like described in this study. As Ojala (2007) pointed out in her study including young environmental volunteers: “it is not the ability to get rid of worry that should be sought after but rather the capacity to face worry, to learn from it, and to use it for constructive actions.” Leaving people alone with their emotions as a consequence to eco-visualizations could lead to negative effects such as reduced levels of self-efficacy or maladaptive coping strategies (Moser and Dilling, 2011; Nabi et al., 2018; Nabi and Myrick, 2019).

## Limitations

This study was part of developing the NIYF methodology further and helped us build a proof-of-concept (PoC) that will be investigated further in future studies. It has successfully shown that the disruptive communication, if used as part of the NIYF structured workshop study can be used to engage children in solving environmental challenges. As an explorative study, aimed at developing the concept further, there are limitations.

The study does give us insight into how children can be included in the plastic pollution issue. It has also given us some initial insights into how children understand—and respond to being exposed to—the problem of plastic pollution as part of a disruptive communication approach. We do not know yet, however, if these results can be generalized over other topics, communities or age groups. We can further not exactly say if the psychological mechanisms we identified throughout the discussions can be verified via quantitative measurements using standardized methods. We have aimed at providing a high degree of transparency in how this workshop was carried out and the conditions around the workshop in order to give the results credibility. Carrying out additional NIYF workshops will allow for generalizability of the workshop results. In further NIYF studies, carried out in other communities and involving multiple age groups, we will also carry out quantitative evaluation that will accompany the workshops in the project. These future workshops will, using a mixed methods approach, allow for generalizable and reliable results. In future studies we will also measure the impact in form of carbon emission reduction with regards to the transition of the local community to plastic neutrality (the overall aim of the Kristiansund municipality’s part of the NIYF main project).

## Conclusion

Our aims to evoke emotions and creative engagement using eco-visualizations as form of disruptive communication have been achieved. We therefore conclude that the NIYF framework may indeed be used as a means to engage children in solving environmental challenges. In comparison to gradual change making processes, disruptive communication offers a promising route forward to tackle the environmental challenges we are facing in the world. Implemented on a larger scale, disruptive communication could function as a wakeup call for immediate action.

We conclude that disruptive eco-visualization can create emotional responses and active engagement amongst children. Our results further show that there are to be three concepts reflecting how children perceive plastic pollution: (1) Emotions related to plastic pollution, (2) Attitudes related to plastic, and (3) Perceptions of plastic pollution. Furthermore, these themes could be linked to eco-anxiety, denial, self-efficacy and cognitive dissonance. Active engagement is a key part of the NIYF methodology as it allows people to channel their emotions into action and thereby, potentially, increase their self-efficacy levels. In the future, we aim to validate this novel methodology across further environmental challenges, communities, and age groups. We also aim to measure impact in form of carbon emission levels as a consequence of the workshops. Thereby we will explore how the full potential of NIYF can be realized.

## Data Availability Statement

The original contributions presented in the study are included in the article/supplementary material, further inquiries can be directed to the corresponding author/s.

## Ethics Statement

Written informed consent was obtained from the individual(s), and minor(s)’ legal guardian/next of kin, for the publication of any potentially identifiable images or data included in this article.

## Author Contributions

EL and IR wrote the manuscript. IN and EL collected all research data. All authors have contributed to the manuscript and approved the submitted version.

## Conflict of Interest

The authors declare that the research was conducted in the absence of any commercial or financial relationships that could be construed as a potential conflict of interest.

## Publisher’s Note

All claims expressed in this article are solely those of the authors and do not necessarily represent those of their affiliated organizations, or those of the publisher, the editors and the reviewers. Any product that may be evaluated in this article, or claim that may be made by its manufacturer, is not guaranteed or endorsed by the publisher.

## Footnotes

1 Richter, I., Pahl, S., Sumeldan, J., Gabe-Thomas, E., Avillanosa, A., and Creencia, L. (under review). Future scenarios as a tool to communicate sustainable development in coastal communities in Palawan, Philippines. *Front. Psychol. Environ. Psychol.*
